# Effects of Milk Yield, Feed Composition, and Feed Contamination with Aflatoxin B1 on the Aflatoxin M1 Concentration in Dairy Cows’ Milk Investigated Using Monte Carlo Simulation Modelling

**DOI:** 10.3390/toxins8100290

**Published:** 2016-10-09

**Authors:** H. J. van der Fels-Klerx, Louise Camenzuli

**Affiliations:** RIKILT Wageningen University & Research, P.O. Box 230, Wageningen 6700 AE, The Netherlands; louise.camenzuli@wur.nl

**Keywords:** transfer, aflatoxins, dairy chain, maize, contamination

## Abstract

This study investigated the presence of aflatoxin M1 (AfM1) in dairy cows’ milk, given predefined scenarios for milk production, compound feed (CF) contamination with aflatoxin B1 (AfB1), and inclusion rates of ingredients, using Monte Carlo simulation modelling. The model simulated a typical dairy farm in the Netherlands. Six different scenarios were considered, based on two lactation and three CF composition scenarios. AfB1 contamination of the CF was based on results from the Dutch national monitoring programme for AfB1 in feed materials from 2000 until 2010. Monitoring data from feed materials used in CF production for dairy cattle in the Netherlands were used. Additionally, AfB1 contamination data from an incident in maize in 2013 were used. In each scenario, five different transfer equations of AfB1 from feed to AfM1 in the milk were used, and 1000 iterations were run for each scenario. The results showed that under these six scenarios, the weekly farm concentration of AfM1 in milk was above the EC threshold in less than 1% of the iterations, with all five transfer equations considered. However, this increased substantially in weeks when concentrations from the contaminated maize batch were included, and up to 28.5% of the iterations exceeded the EC threshold. It was also observed that an increase in the milk production had a minimal effect on the exceedance of the AfM1 threshold due to an apparent dilution effect. Feeding regimes, including the composition of CF and feeding roughages of dairy cows, should be carefully considered based on the potential AfM1 contamination of the farm’s milk.

## 1. Introduction

Aflatoxin B1 (AfB1) is a genotoxic and carcinogenic mycotoxin that is produced by fungi, in particular *Aspergillus flavus* and *Aspergillus parasiticus.* AfB1 can be metabolized to aflatoxin M1 (AfM1) by cows, sheep, and goats. Like AfB1, AfM1 is also considered to be genotoxic and carcinogenic to animals and humans [1]. Elevated concentrations of AfB1 in feed result in elevated levels of AfM1 in milk and milk products. Due to their toxic effects on human and animal health, the presence of AfB1 and AfM1 in foodstuffs is strictly regulated within the European Union (EU). Regulation (EC) No 1881/2006 sets the maximum levels for aflatoxin M1 in dairy products, i.e., 0.05 µg/kg for raw milk, heat-treated milk, and milk for the manufacture of milk-based products. In addition, a maximum level for AfB1 in all feed materials was set at 0.02 mg/kg, as well as for compound feed for cattle, sheep, and goats, with the exception of dairy cattle, dairy sheep, and dairy goats at 0.005 mg/kg (Directive 2002/32/EC, Consolidated version 27 February 2015), all relative to a feed with a moisture content of 12%.

Historically, aflatoxins have been mainly found in products originating from countries with tropical weather conditions favourable for the growth of *Aspergillus* spp., like India, Brazil, and Colombia. However, during the last decade, serious contaminations of maize with aflatoxins have been reported in southern Europe. These include maize grown in 2003 in Italy [2] and maize from the 2012 harvest from the Balkan region [3]. Since climate change is increasingly affecting the formation of mycotoxins in Europe [4], these recent incidents are most likely not the last cases of aflatoxins in maize for European farmers.

Maize is a commonly used ingredient for feeding dairy cows. Feed manufacturers produce feed by mixing and grinding maize and other feed ingredients. In recent decades, the maize consumption of dairy cows has increased due to the prices of raw materials, compound feed composition, and the increase in the amount of concentrated feeds in diets. For example, in the Netherlands, the inclusion rates of maize in compound feed for dairy cows has increased from approximately 1% to 18% in the period 2010–2013 [5]. Additionally, the milk production of dairy cows is steadily increasing, which will coincide with an increased intake of feed, particularly compound feed, by cows.

The abovementioned developments have possibly increased the total exposure of dairy cows to aflatoxins, which might in turn lead to a higher probability of dairy cows’ milk to be contaminated by AfM1.

The aim of this study was to estimate the AfM1 contamination in dairy cows’ milk, using transfer modelling under multiple scenarios of compound feed composition, feed contamination with AfB1, feed consumption, and milk yield. The inclusion rate of maize in compound feed and the contamination of maize by AfB1 were modelled by Monte Carlo simulations, and hence the AfM1 concentration in dairy cows’ milk with the intake distribution could be investigated. Monte Carlo simulation is a computerized mathematical technique that is often used for quantitative analyses and decision-making. The strength of this simulation technique is that it provides the decision-maker with a range of possible outcomes together with their probabilities.

## 2. Results

### 2.1. Transfer of AfB1 in Feed to AfM1 in Milk

The transfer of AfB1 in feed to AfM1 in milk was modelled for a typical Dutch dairy farm using transfer modelling combined with Monte Carlo modelling (1000 iterations) under six different scenarios. Namely, three compound feed (CF) composition scenarios, under two different milk yield scenarios (normal and extreme lactation). Five different transfer equations (Materials & Methods Table 4) obtained from literature were used to model the transfer from AfB1 in feed to AfM1 in milk. AfB1 contamination in feed ingredients was modelled in line with the results from the Dutch monitoring data, except for weeks 25 and 26, during which data from a contaminated maize batch were used [3].

Model output is weekly resolved, and for each week, the percentage of simulations (from the 1000 iterations) which resulted in an exceedance of the EC limit of 0.05 µg/kg for AfM1 in milk was calculated. For each scenario, the maximum of these weekly percentage exceedances are shown in Table 1. The week with the maximum percentage exceedance coincided with the use of highly contaminated maize in compound feed. In Table 1, the results of the week with the highest percentage exceedance rate without using the contaminated maize batch are also shown (in italics). Less than 1% of all the weekly simulations were above the EC limit for AfM1 in milk, when only monitoring data were used.

The transfer equation from Veldman et al. [7] resulted in the highest percentage of simulations above the EC limit, with an exceedance in 28.3% of the weekly simulations. The use of a low-protein compound feed (CF composition Scenario 2) resulted in the highest percentage of simulations above the EC limit for all transfer equations. This is in line with the fact that the low-protein compound feed has a high maize inclusion rate. With most of the transfer models, however, no clear differences could be observed in the amount of simulations above the EC threshold between the two lactation scenarios. In the extreme lactation scenario, all cows start lactating at the same time, as opposed to having different cows starting their lactation cycle on different weeks (normal lactation). Under the extreme lactation scenario, even when the cows consume highly contaminated feed in the same weeks (weeks 25 & 26) during their lactation peak, a higher transfer rate of AfM1 has not resulted in an increased exceedance rate due to the high volume of milk produced in the farm, and even a decrease when using the model of Van Eijkeren et al. [9]. In our model, the farm weekly milk production in week 25 amounts to 12,000 kg under the normal lactation milk yield scenario. The extreme lactation scenario resulted in 25% higher milk yield in the same week. Hence, the reason for the similar number of simulations above the EC threshold for both lactation scenarios is probably due to dilution. This dilution effect is most clear for the scenarios using the transfer equation provided in the EFSA opinion [10] from Pettersson [11], where the maximum weekly percentage of simulations above the EC limit is lower under the extreme lactation scenarios, than under the normal lactation scenarios. In the equation from Pettersson [11], the concentration of AfM1 in milk is only dependent on the total AfB1 intake (Materials & Methods Table 4). In the remaining scenarios, the transfer is dependent both on the total AfB1 intake and on the milk yield, and hence this dilution effect is less obvious due to the interaction between the intake and milk yield. Consequently, when comparing the percent of simulations above the EC threshold of a scenario under normal lactation and the corresponding scenario under extreme lactation, the difference is minimal. This implies that a higher milk production has an overall minimal effect on the concentration of AfM1 in the milk of the farm. When using the model from Van Eijkeren et al. [9], the dilution effect is such that with the extreme lactation the probability of AfM1 exceedance is even lower than with the normal lactation.

For each weekly model output, the mean concentration of AfM1 in the milk produced at the farm over 1000 iterations was also calculated. The maximum values for all these weekly mean concentrations are presented in Table 2. For all the scenarios modelled, the maximum weekly mean falls within the EC limit for AfM1 in milk. In some of the considered scenarios, this maximum of the weekly mean concentrations can reach up to 0.04 μg/kg (Table 2). Concentrations of AfM1 in milk as high as 0.32 µg/kg were modelled, which is 6.4 times the EC limit. Notably, this high concentration was calculated under CF Scenario 3, using the transfer equation from Veldman et al. [7]; it also coincided with the weeks when contaminated maize was used.

### 2.2. Effect of Milk Yield and Feed Intake

Additional scenarios other than the standard scenarios were modelled in order to investigate the interaction between high/low yielding cows and high/low feed intake. The yearly milk yield per cow as modelled under the standard scenarios (Section 2.1) was equivalent to 9111 kg, with high and low yielding cows producing 11,845 and 6378 kg per year, respectively. Similar to the standard scenarios, each week, the percentage of simulations above the EC limit of 0.05 µg/kg for AfM1 in milk was calculated. The maximum values of these weekly percentages for each additional scenario are shown in Table 3. In comparison with Table 2 (specifically CF composition 1 under normal lactation), the high feed (HF) scenarios resulted in a higher weekly percent of simulations exceeding the EC limit. This is true for both the high milk yield (HY) and the low milk yield (LY) cows; however, the effect is larger for the high milk yield scenarios. The low feed (LF) scenarios resulted in a lower percent of simulations exceeding the EC limit, even for simulations with a high milk yield (HY). Additionally, the maximum weekly percent of simulations above the EC limit also coincided with the weeks when contaminated maize was used. If these weeks are excluded, the maximum percent of simulations above the limit is drastically reduced (percent shown in italics).

The mean AfM1 concentration as modelled for the whole farm was calculated every week as the mean of the 1000 iterations. The maximum of these weekly mean AfM1 concentrations in milk followed a similar trend when compared to the standard feed/standard milk yield scenario. The maximum of the weekly mean concentrations in all four scenarios did not exceed the EC limit; however, the highest farm AfM1 concentration modelled was 0.22 µg/kg under the high feed and low yield scenario (HF_LY) when using the equation by Veldman et al. [7] during the weeks with contaminated maize.

The final scenario modelled included corn silage as an additional source of AfB1 in the daily diet. The AfB1 contamination in corn silage was set at 1 µg/kg, being the limit of quantification. When compared to the corresponding scenario without the inclusion of contaminated silage (CF composition 1 under normal lactation), the percentage of simulations above the EC limit for AfM1 in milk is approximately doubled. Nevertheless, excluding weeks 25 and 26, the percent of simulations above the AfM1 limit is still less than 1%. This shows that this additional contamination at a low level does not contribute significantly to raising the daily AfB1 intake above the threshold required to exceed the AfM1 threshold in milk.

### 2.3. Effect of the Variation in the Transfer Equations

Under the same milk yield scenario, and the same compound composition, the equation used for the transfer of AfB1 in feed to AfM1 in milk played an important role. The exceedance percentage was up to six times higher, depending on which transfer equation is used. The equations established by Masoero et al. [6], Veldman et al. [7], Britzi et al. [8], and Van Eijkeren et al. [9] relate the concentration of AfM1 in milk to the daily milk yield and to the daily intake of AfB1. The equation from Pettersson in 1998 [11]—presented in the EFSA opinion in 2004 [10] on AfB1 in animal feed—related the AfM1 in milk to the daily intake of AfB1, irrespective of the daily milk yield. The transfer rate in the equations from Masoero et al. [6] and Van Eijkeren et al. [9] depends similarly on milk yield, and hence similar results were obtained when using these two transfer equations. On the other hand, the equation from Veldman et al. [7] results in an overall higher transfer rate. Hence, the modelled concentration of AfM1 in milk when using the equation from Veldman et al. [7] will always be the highest. The equation from Britzi et al. [8] has the highest dependence of transfer rate on milk yield. Hence, the modelled concentration of AfM1 in milk will vary greatly depending on the phase in the lactation cycle. On the contrary, in the model described by Van Eijkeren et al. [9] the dependence is very small.

## 3. Discussion 

This study aimed at investigating the potential exceedance of the maximum limit for AfM1 concentrations in milk, under different scenarios for AfB1 contamination of feed ingredients, inclusion rates of ingredients in compound feed, milk production, and transfer rates. Monte Carlo simulation modelling was used to investigate the AfM1 contamination of milk for the wide range of situations that realistically occur in practice. The results show that milk production of the farm and milk yield appeared to have minimal effects on the outcomes; a higher milk production did not result in increased concentrations of AfM1 in the farm milk, due to a potential dilution effect. While, according to transfer equations, a higher milk yield will result in a higher transfer rate, the concentration in the milk produced on the farm changes only slightly due to an increased milk production.

The Monte Carlo approach was applied to simulate the AfB1 contamination in each individual feed ingredient, using monitored mean and standard deviation AfB1 concentrations in the respective feed ingredient monitored over a 10-year period. Thus, the monitoring results were used to fit a distribution for AfB1 in each feed ingredient, and this distribution was in turn used as an input in the Monte Carlo simulation. The same approach was used to simulate the compound feed composition for Scenario 3, provided a known minimum and maximum percentage inclusion for each ingredient. This approach allowed us to simulate 1000 different contamination/composition pairs for the daily feed intake for AfB1, changing every two weeks. With 10-year monitoring data of AfB1 contamination in feed ingredients and the guidelines available for feed composition, less than 0.6% of all the simulations for individual two-week periods were above the EC limit for AfM1 concentration in milk, with a maximum concentration of 0.08 µg/kg (over all five transfer equations). This resulted from either a high inclusion rate of maize with an AfB1 concentration below the EC limit in compound feed, or from a low inclusion rate of maize above the EC limit for AfB1 in the compound feed. Nevertheless, it can be concluded that given the current practices, the probability of exceeding the EC limit of AfM1 in dairy milk is very low. In our model we also tested the effect of corn silage contaminated with AfB1 on the AfM1 content in milk. The contamination of corn silage was set to the limit of quantification of AfB1. With an inclusion rate of corn silage in the dairy cow diet of 27% and a low AfB1 contamination level, a minimal change in the AfM1 content in milk was observed.

Our model concludes that under current inclusion rates of ingredients in compound feed and current AfB1 levels in feed ingredients, the probability of exceeding this EC limit is very low, and only a highly contaminated batch would be cause for concern. When considering the same lactation scenario and the same compound feed composition scenario, an increase in the milk yield and the daily feed intake by a factor of 1.3 resulted in a maximum increase of 0.2% in the probability of exceeding the EC limit in milk. Hence, using the current guidelines for compound feed composition, and the current limits on AfB1 in feed materials, an increase in milk yield does not appear to increase the probability of exceeding the limit of AfM1 in milk.

In 2013, a contaminated shipment of maize intended for feed materials was imported into the Netherlands [3]. The batch had a mean AfB1 concentration of 50.2 (±36.1) µg/kg, which is much higher than the EC legal limit for using maize as an ingredient (being 0.02 mg/kg at a moisture content of 12%). This batch was, however, not found to be contaminated during regular monitoring and was used for the production of a compound feed for dairy cattle. Such a batch was included in our transfer model, also using Monte Carlo simulations for the simulation of the AfB1 contamination of the feed ingredients and for the inclusion rate of each ingredient for a two-week period (weeks 25 and 26). Under normal lactation, 5%–28% of the simulations exceeded the EC limit for AfM1 in milk in the respective weeks. However, the probability, when including this contaminated feed in our model, was considerably dependent on the transfer equation used.

The transfer rate used to set the EC legislative limit in feed for dairy cows was 1%–2%. However, several studies found higher transfer rates of aflatoxins in cows with a higher milk yield [6,7,12] and in early/mid-lactating cows [7,8,13,14,15]. This was incorporated in our model through a 45-week lactation cycle, with milk yield varying through the cycle. The transfer rate varied, possibly due to differences between the metabolisms of the cows, the milk yield, and the source of contamination. In fact, the source of the contamination, milk yield, and cow breed in the studies available varied. Veldman et al. [7] used contaminated groundnut meal, Britzi et al. [8] and Masoero et al. [6] used contaminated corn meal, and the model by Van Eijkeren et al. [9] was fitted to data by Frobish et al. [16] using contaminated cottonseed. Concerning breed, Britzi et al. [8] carried out their study on Israeli Holstein cows (high-yield cows with an average yield of 11,400 kg _milk_/cow), Masoero et al. [6] carried out their study on Holstein cows, and Veldman et al. [7] did not specify which cows were used. The maximum transfer rate from Veldman et al. [7] and Britzi et al. [8] was about 6%; however, in the model set up by Van Eijkeren et al. [9], the maximum transfer rate was 3.2%. When the model set up by Van Eijkeren et al. [9] was applied to the results from Masoero et al. [6] and Veldman et al. [7], the model did not agree with the data. The rate of exceedance of the EC threshold varied considerably depending on the equation used. However, it is unclear which of these equations is most suitable within our model, and hence all should be considered.

## 4. Conclusions 

Given the ranges in the available transfer equations, only when a highly contaminated batch was included in the model was there a high possibility of exceeding the EC threshold of AfM1 in milk. Additionally, this depended on the inclusion rate of maize in the compound feed. Regarding the increased use of maize in feed for dairy cows, and the increasing milk yield, we hypothesised higher concentrations of AfM1 in dairy cows’ milk. This study showed that under current practise and for the current limits on feed ingredients, an increased milk yield alone will not affect the contamination rate of milk with AfM1. However, to some extent, an increased use of maize in compound feed, combined with a higher contamination of maize with AfB1, will indeed increase the probability of exceedance of the EC limit for AfM1 in milk. Therefore, composition of compound feeds for dairy cows should be carefully performed, and should include information on the potential of AfB1 contamination of the ingredients used, as was shown by Van der Fels-Klerx and Bouzembrak [17], so as to comply with the EC limit for AfB1 in the final feeds.

## 5. Materials and Methods

### 5.1. Model

A simulation model was developed in order to estimate the distribution of AfM1 concentrations in dairy milk in a typical farm in the Netherlands. The model was developed in MATLAB R2015b. The model outline, given below, is based on a Monte Carlo simulation (1000 iterations) of the daily intake of aflatoxin B1 from compound feed. Monte Carlo simulation was used to assess the wide variety of possibilities that realistically occur in each of these steps, i.e., in compound feed composition, contamination of the feed ingredients with AfB1, milk production, and transfer of AFB1 to AFM1 in milk.

$$\begin{array}{ccccccc} \begin{array}{c} \text{Compound feed composition scenarios}\\ \&\\ \text{[AfB1] in compound feed ingredients}\;i \end{array} & \to & \text{Distribution of daily intake of AfB1} & + & \begin{array}{c} \text{Different scenarios for milk yield}\\ \&\\ \text{Different transfer equations} \end{array} & \to & \text{Distribution of [AfM1] in milk} \end{array}$$

In the model, it is assumed that all the cows in the farm are housed indoors throughout the year. The modelled farm was set up based on the survey by Driehuis et al. [18] among 24 dairy farms across the Netherlands. An average herd size of 69 cows was assumed, with an average total daily feed intake (and standard deviation) of 18.7 (1.3) kg _DM_/cow, of which 4.3 (0.2) kg _DM_/cow is compound feed.

The milk yield per cow was modelled according to an incomplete gamma model developed by Wood [19] and discussed in Olori et al. [20]. The lactation period per cow was set to 45 weeks, followed by a four-week dry period. The milk yield for the whole farm was modelled in two different milk yield scenarios, namely (1) an individual cow started a new lactation cycle each week (normal lactation scenario); and (2) as an extreme scenario, all cows started the lactation cycle on the same day (extreme lactation scenario).

The concentration of AfM1 in milk ([AfM1]_milk_) can be modelled through several equations; Table 4 presents the equations published in the scientific literature. Masoero et al. [6],Veldman et al. [7], and Britzi et al. [8] experimentally related the concentration of AfM1 in milk to the total intake of AfB1 (total intake_AfB1_) and the daily milk yield (Y_milk_). Van Eijkeren et al. [9] estimated the concentration of AfM1 at steady state, also from the total intake of AfB1 and the daily milk yield. The EFSA opinion from 2004 [10] on AfB1 in animal feed uses the equation from Pettersson [11], which is based on a collection of different experimental studies.

### 5.2. Input Data

#### 5.2.1. AfB1 Contamination in Compound Feed

Data on aflatoxin concentrations in maize and other feed ingredients used in compound feed production for dairy cows, over the years 2000–2010, were extracted from the national monitoring database named KAP (Quality of Agricultural Products) in the Netherlands. These included 3427 records in total. All measurements below the limit of detection (LOD) of 1 µg/kg are recorded as zero in the database. A summary table of the data is presented in Table 5. In addition to the information from the KAP database, data from a recent incident in 2013 [3] of maize from the Balkan region, being contaminated above the maximum level of 20 µg/kg, were also considered. These data were from a shipment of maize dated from March 2013, intended for feed production in the Netherlands, as described by De Rijk et al. [3].

#### 5.2.2. Compound Feed (CF) Composition

Data available for compound feed composition in the Netherlands for lactating dairy cows, including the recipe and the rate to which individual ingredients are used in the compound feed, are presented in Table 6 [21]. These include the mean rate of use of each ingredient in 2014, for each of high- and low-protein compound feed for dairy cattle, as well as the range (minimum–maximum) to which each ingredient is used. Based on these data, three different scenarios for compound feed composition were defined. In the high- and low-protein scenarios, the fixed percentage of each ingredient was used in each simulation, whereas in the third scenario, the minimum and maximum ranges were used to simulate 1000 different compound feed compositions, with composition percentage within the suggested range.

The data from Table 4 and Table 5 were combined to calculate the total intake of AfB1 from compound feed (CF) composed of ingredients *i*, using the equation below. It is assumed that a compound feed consists of 85% dry matter (DM) [22].

$${\mathrm{Total daily intake}}_{\mathrm{AfB}1}=\frac{\mathrm{daily feed intake}\cdot \%\;\mathrm{CF in feed}}{0.85}\cdot \left(\sum_{n=1}^{i}{\left[\mathrm{AfB}1\right]}_{i}\cdot \%\;i\;\mathrm{in CF}\right).$$

**Figure 1:**
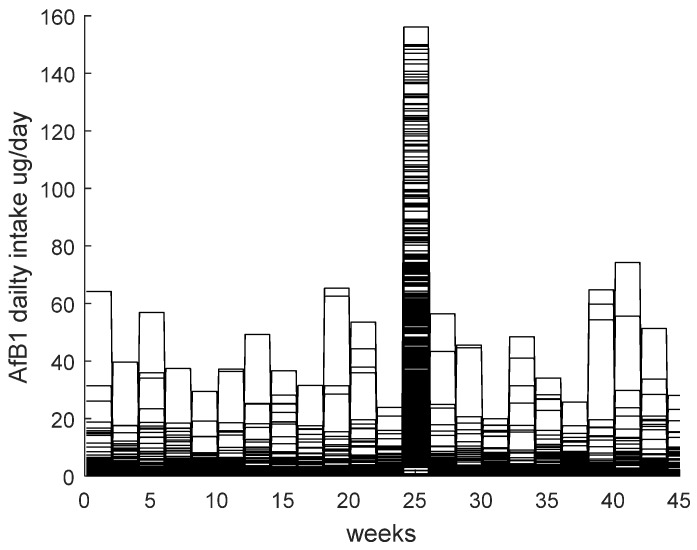
Daily intake of AfB1 from dairy cows’ compound feed over for 1000 simulations over the whole lactation period.

Additionally, it is assumed that a fresh batch of compound feed is used every two weeks and that all cows eat the same amount of feed, meaning that over a two-week period the total daily intake of AfB1 is constant for all cows. Figure 1 shows an example of the total daily intake for the 1000 iterations, changing every two weeks.

### 5.3. Scenarios

The Monte Carlo simulation model was run for each of the six different basis scenarios, with two lactation scenarios by three compound feed composition scenarios, using each of the five different transfer equations (Table 4). In each scenario, 1000 iterations were used. AfB1 contamination data from KAP were used for all weeks, except for weeks 25 and 26, when data from the contaminated maize batch was used. Under the extreme lactation scenario, all cows have maximum milk yield during weeks 25 and 26, hence coinciding with the fortnight of contaminated maize.

In order to investigate the interaction between high/low yielding cows and high/low feed intake, additional scenarios were also modelled. High and low yielding cows were modelled as 1.3 and 0.7 times, respectively, of the milk yield used in the scenarios above. In accordance with the average total feed intake (and standard deviation) presented by Driehuis et al. [18], total daily feed intake for high and low feeding cows is modelled as 23 (high feed, HF) and 14.5 kg·_DM_/cow (low feed, LF), respectively. These four feed/milk yield pairs were modelled with compound feed Scenario 1 (high-protein diet), and the normal lactation scenario. The final scenario modelled, also the worst case scenario, assumed contaminated corn silage at the limit of quantification (LOQ) of AfB1. Information from the KAP database shows that the contamination of corn silage in the Netherlands from 2001 until 2010 is below the LOQ, and hence setting the contamination at the LOQ represents the worst case scenario under current conditions. The percentage of corn silage in the total daily feed intake was set at 27% [18], with a contamination of AfB1 of 1 µg/kg and an assumed dry matter content of 30% [22].

## Figures and Tables

**Table 1:** Maximum weekly percentage * of simulations above the threshold of AfM1 in milk from the whole farm. Numbers in italics represent the maximum percent of simulations in all weeks excluding weeks 25 and 26 (when contaminated maize was used).

CF Composition Scenario	Milk Yield Scenario	Transfer Model									
		Masoero et al. [6]		Veldman et al. [7]		Britzi et al. [8]		Van Eijkeren et al. [9]		Pettersson from EFSA Opinion [10]	
1	normal	4.9	*0.0*	16.5	*0.3*	7.0	*0.1*	6.0	*0.0*	12.5	*0.2*
	extreme	4.8	*0.0*	16.3	*0.3*	8.9	*0.1*	4.7	*0.1*	12.3	*0.1*
2	normal	11.2	*0.0*	28.3	*0.5*	15.0	*0.1*	13.7	*0.1*	23.0	*0.2*
	extreme	11.9	*0.1*	28.5	*0.3*	17.3	*0.1*	11.2	*0.1*	22.8	*0.2*
3	normal	8.6	*0.3*	20.9	*0.6*	11.6	*0.3*	10.3	*0.3*	16.6	*0.5*
	extreme	7.5	*0.1*	18.9	*0.3*	11.2	*0.2*	7.2	*0.2*	14.8	*0.2*

* calculated as (the number of simulations in which the farm milk concentration is above 0.05 µg/kg) /1000 simulations × 100.

**Table 2:** Maximum of weekly mean AfM1 concentrations (µg/kg) in milk from the whole farm (over all iterations) *.

CF Composition Scenario	Milk Yield Scenario	Transfer Model				
		Masoero et al. [6]	Veldman et al. [7]	Britzi et al. [8]	Van Eijkeren et al. [9]	Pettersson [11] from EFSA Opinion [10]
1	normal	0.015	0.028	0.018	0.017	0.029
	extreme	0.015	0.028	0.020	0.015	0.029
2	normal	0.022	0.040	0.026	0.025	0.037
	extreme	0.022	0.041	0.029	0.022	0.037
3	normal	0.018	0.033	0.021	0.020	0.032
	extreme	0.017	0.031	0.022	0.017	0.031

* Including the contaminated batch in weeks 25 & 26. Please note that the maximum of the highest of the weekly mean concentrations is always seen with the contaminated batch.

**Table 3:** Maximum weekly percentage * of simulations above the AfM1 threshold in milk from the whole farm (HF = High Feed; LY = Low Yield). Numbers in italics represent the maximum percent of simulations in all weeks excluding weeks 25 and 26 (when contaminated maize was used).

Feed and Yield Scenario	Masoero et al. [6]		Veldman et al. [7]		Britzi et al. [8]		Van Eijkeren et al. [9]		Pettersson [11] from EFSA Opinion [10]	
HF_HY	8.7	*0.1*	23.4	*0.5*	14.5	*0.3*	6.9	*0.0*	16.7	*0.3*
HF_LY	6.1	*0.1*	22.2	*0.4*	10.0	*0.1*	15.0	*0.3*	17.2	*0.3*
LF_HY	1.8	*0.0*	11.3	*0.1*	6.1	*0.0*	1.0	*0.0*	7.3	*0.1*
LF_LY	0.6	*0.0*	10.6	*0.1*	2.7	*0.0*	5.4	*0.0*	8.0	*0.1*
contaminated silage	8.6	*0.1*	33.0	*0.6*	13.6	*0.3*	12.0	*0.2*	23.8	*0.4*

* calculated as (the number of simulations in which the farm milk concentration is above 0.05 µg/kg) /1000 simulations × 100.

**Table 4:** Equations used for modelling the transfer of AfB1 in feed to AfM1 in dairy milk.

Equation	Source
${\left[\mathrm{AfM}1\right]}_{\mathrm{milk}}\left({\mu g}_{\mathrm{AfM}1}/{\mathrm{kg}}_{\mathrm{milk}}\right)={\mathrm{Total}\;\mathrm{intake}}_{\mathrm{AfB}1}\cdot Y_{\mathrm{milk}}^{-1}\cdot \left[Y_{\mathrm{milk}}\cdot 0.13-0.26\right]/100$	[6]
${\left[\mathrm{AfM}1\right]}_{\mathrm{milk}}\left({\mu g}_{\mathrm{AfM}1}/{\mathrm{kg}}_{\mathrm{milk}}\right)={\mathrm{Total}\;\mathrm{intake}}_{\mathrm{AfB}1}\cdot Y_{\mathrm{milk}}^{-1}\cdot \left[Y_{\mathrm{milk}}\cdot 0.077-0.326\right]/100$	[7]
${\left[\mathrm{AfM}1\right]}_{\mathrm{milk}}\left({\mu g}_{\mathrm{AfM}1}/{\mathrm{kg}}_{\mathrm{milk}}\right)={\mathrm{Total}\;\mathrm{intake}}_{\mathrm{AfB}1}\cdot Y_{\mathrm{milk}}^{-1}\cdot \left[0.5154\cdot e^{Y_{\mathrm{milk}}\cdot 0.0521}\right]/100$	[8]
${\left[\mathrm{AfM}1\right]}_{\mathrm{milk}}\;\left({\mu g}_{\mathrm{AfM}1}/{\mathrm{kg}}_{\mathrm{milk}}\right)={\mathrm{Total}\;\mathrm{intake}}_{\mathrm{AfB}1}\cdot \left[Y_{\mathrm{milk}}\cdot 0.032\right]\cdot {\left(17+Y_{\mathrm{milk}}\right)}^{-1}$	[9]
${\left[\mathrm{AfM}1\right]}_{\mathrm{milk}}\left({\mathrm{ng}}_{\mathrm{AfM}1}/{\mathrm{kg}}_{\mathrm{milk}}\right)={\mathrm{Total}\;\mathrm{intake}}_{\mathrm{AfB}1}\cdot 0.787+10.95$	[10]

**Table 5:** Summary data for AfB1 concentration (µg/kg) in individual feed ingredients.

Ingredient	# of Records	Min. [AfB1]	Max. [AfB1]	Mean [AfB1]	Std. Deviation
Wheat ^1^	346	0	1.4	0.019	0.148
Barley ^1^	155	0	1.5	0.016	0.144
Corn ^1^	768	0	115	0.653	5.52
Triticale ^1^	24	0	1.9	0.079	0.388
Rye ^1^	23	0	0	0	0
Soybean meal ^1^	751	0	5.00	0.0395	0.269
Sunflower scrap ^1^	136	0	7.5	1.09	1.72
Palm kernel ^1^	484	0	26.0	0.515	3.08
Rapeseed scrap ^1^	71	0	0	0	0
Corn gluten feed ^1^	517	0	42.0	0.461	2.03
Flour ^1^	2	0	0	0	0
Citrus pulp ^1^	114	0	1.5	0.022	0.168
Dried beet pulp ^1^	34	0	0	0	0
Molasses ^1^	2	0	0	0	0
Contaminated maize ^2^	72	6.2	168	50.2	36.1

^1^ Data from the KAP database, ^2^ Data from De Rijk et al. [3].

**Table 6:** Feed composition (%) under a high-protein (Scenario 1) and a low-protein diet (Scenario 2). The general guidelines for feed composition are provided in Scenario 3 as the minimum and maximum percentages for each feed material.

Feed Ingredients	High-Protein	Low-Protein	Minimum	Maximum
CF composition scenario	1	2	3	
*Total grains*			*20*	*60*
Wheat	5	6	0	35
Barley	0	2.39	0	0
Corn	10.24	15.06	0	35
Triticale	1.2	1.9	0	15
Rye	0	1	0	15
Soybean meal	14.96	0.23	0	30
Sunflower seed meal	4.5	3.83	0	25
Palm kernel	15.01	15	0	20
Rapeseed meal	7.94	5.54	0	30
Corn gluten feed	3.67	1	0	30
Flour	0	0.04	0	20
Dried beet pulp	0.08	7.86	0	40
Citrus pulp	0	3.37	0	25
Molasses	1.5	1.55	0	10
